# Validation of SCORE2 on a sample from the Russian population and adaptation for the very high cardiovascular disease risk region

**DOI:** 10.1371/journal.pone.0300974

**Published:** 2024-04-17

**Authors:** Gleb E. Svinin, Vladimir A. Kutsenko, Svetlana A. Shalnova, Elena B. Yarovaya, Asiia E. Imaeva, Yulia A. Balanova, Anna V. Kapustina, Galina A. Muromtseva, Oxana M. Drapkina

**Affiliations:** 1 Department of Epidemiology of Non-Communicable Diseases, National Medical Research Center for Therapy and Preventive Medicine, Moscow, Russia; 2 Department of Probability Theory, Faculty of Mechanics and Mathematics, Lomonosov Moscow State University, Moscow, Russia; 3 National Medical Research Center for Therapy and Preventive Medicine, Moscow, Russia; Vellore Institute of Technology, INDIA

## Abstract

SCORE2 (Systematic COronary Risk Evaluation 2) is a risk assessment scale for cardiovascular events, presented in 2021 by the European Society of Cardiology. Both for training and validation of the SCORE2 model, representative samples from the Russian population were not used. Therefore, we aimed to validate SCORE2 on a such sample. For this purpose, we used a sample from the ESSE-RF epidemiological study consisting of 7251 participants aged 40–69 years without history of CVDs. We performed the validation by comparing SCORE2 risk estimates for ESSE-RF participants with the observed incidence of cardiovascular events in the study, adjusted for event information losses. The validation demonstrated that SCORE2 risk estimates were accurate for Russian men and inaccurate for Russian women. Together with the quantitative assessment of risk, SCORE2 offers its interpretation in terms of 10-year CVD risk group: low-moderate, high, and very high. For Russian men we considered the original interpretation of the SCORE2 estimates to be questionable because almost none of the men would be categorized as having “low-to-moderate” 10-year CVD risk. This problem would be typical for all countries of the very high CVD risk region. Therefore, we proposed a new interpretation of the SCORE2 risk estimates for men from the very high risk region. According to the proposed interpretation, the fraction of men in ESSE-RF in “low-to-moderate” 10-year CVD risk increased from 2% to 18% and the fraction of men in “very high” CVD risk decreased from 63% to 20% as compared to the original interpretation. The proposed interpretation would allow a more personalized approach to CVD treatment and optimize the burden on primary healthcare in the very high risk region countries.

## Introduction

One of the most important achievements of clinical epidemiology is the development of risk-prediction models. The European Society of Cardiology (ESC) recommends the use of risk-prediction models to improve healthcare and prevention across the population [1]. The main goal of the models is to identify people at increased risk of cardiovascular diseases (CVD), who might receive the greatest benefit from a preventive intervention [2]. The basis for the development of all future prognostic models was formed in the Framingham Heart Study [3].

The main issue that accompanies any risk assessment model is the accuracy of its predictions. The Framingham model of cardiovascular risk assessment was proved to be quite good in certain conditions and populations [4], but not accurate enough in other populations [5], especially European ones. That formed the basis for the creation of the European Risk Assessment Model SCORE [6]. SCORE provides a direct evaluation of 10-year fatal cardiovascular risk in a format suited to the constraints of clinical practice [6]. In particular, SCORE has been introduced and actively used in Russia for 20 years. It turned out to be very useful in practice since it provided an intuitive way to direct a patient’s treatment strategy [7].

In turn, SCORE has two weaknesses. The first one is that SCORE predicts the risk of fatal cardiovascular (CV) events only. The second one is that SCORE was developed from cohorts recruited before 1986. Both of these aspects were resolved in a new model for predicting cardiovascular risks–SCORE2.

SCORE2 (Systematic COronary Risk Evaluation 2) is a risk assessment scale for cardiovascular events, presented in 2021 by the European Society of Cardiology [2]. SCORE2 evaluates the risk of a CV event over a 10-year period for people aged 40–69 years without a history of cardiovascular disease (CVD), chronic kidney disease, diabetes mellitus (DM), or familial hypercholesterolemia. Gender, age, smoking status, systolic pressure, total cholesterol, and HDL cholesterol are the risk factors in the SCORE2 model [2]. SCORE2 also provides the interpretation of risk estimates in terms of 10-year CVD risk groups: low-to-moderate, high, very high.

SCORE2 has a potential limitation for countries of the very high CVD risk region: for model training representative cohorts from this region were not used. Moreover, for validation within the very high CVD risk region, cohorts from only two cities (Kaunas, Lithuania and Novosibirsk, Russia from the HAPIEE Study) were used. These two cohorts could be hardly considered representative of the entire very high CVD risk region, thus we concluded that SCORE2 was not sufficiently validated for use in the very high CVD risk region [8].

One more questionable aspect to be mentioned is that according to the simplified SCORE2 estimation chart presented in the original article on SCORE2, no men from the very high CVD risk region would be considered at “low-to-moderate 10-year CVD risk” at all [2]. In other words, according to the SCORE2 estimation chart all men from countries of the very high CVD risk region would need to consider some treatment of CVD risk factors. Such strategy is hardly sensible and, if applied, would paralyze the healthcare systems of those countries.

The aim of our study was to evaluate the accuracy of SCORE2 risk estimates for the Russian population and develop an adapted interpretation of SCORE2 risk estimates for clinical practice in Russia and other countries of very high risk region.

## Materials and methods

### Materials

In our work we used data from the following three sources: the epidemiological study ESSE-RF (Epidemiology of Cardiovascular Diseases and their Risk Factors in Some Regions of the Russian Federation); the Moscow part of WHO MONICA Project (Multinational Monitoring of Trends and Determinants of Cardiovascular Diseases); Russian Fertility and Mortality Database (RusFMD).

#### ESSE-RF

Data on frequency of CV events in the Russian population for 2012–2019 were obtained from the ESSE-RF epidemiological study. The recruitment period for ESSE-RF was from September 15^th^, 2012, to June 10^th^, 2014. The response rate of the study was about 80%. The total number of ESSE-RF participants was 18037. For the analysis data from 7251 individuals aged 40–69 years without history of CVD and/or diabetes mellitus 2 type (all according to SCORE2 inclusion criteria) were used. The considered follow up period was 7 years (2013–2019). The period of 2020–2022 was not included into consideration in order to avoid the impact of the COVID-19 pandemic on the analysis. The information about CV events (CVD death, nonfatal myocardial infarction (MI) and/or acute cerebral circulation disorder) and non-CVD deaths among the study participants was collected during the follow up period. In total 234 (3.2%) CV events (fatal or non-fatal) and 135 (1.9%) competing events (non-CVD death) were obtained.

*ESSE-RF ethics statement*. General protocol of the ESSE-RF study has been published previously [9] and the study itself has been registered at clinicaltrials.gov (NCT02449460). The study was approved by three ethics committees: National Medical Research Center for Therapy and Preventive Medicine, Russian Cardiology Research-and-Production Complex, and Federal Almazov North-West Medical Research Centre. The ESSE-RF study was carried out in accordance with the ethical provisions of the Declaration of Helsinki and the National Standard of the Russian Federation “Good Clinical Practice (GCP)” GOST R52379- 2005. In order to comply with the above-mentioned laws, as well as Article 93 of Federal Law No. 323-FZ of November 21, 2011 "On the Fundamentals of Health Protection of Citizens of the Russian Federation", each subject signed a written consent to the processing of their personal data for the purposes of the study.

#### Moscow MONICA

The Moscow part of the WHO MONICA project 1988–1998 (Moscow MONICA) [10] was used as a reference epidemiological study for ESSE-RF. The total number of Moscow MONICA participants was 2420. For the analysis data of 1663 individuals aged 40–64 were used. The considered follow up period for every participant was 7 years. Information about 141 (8.5%) fatal events among the study participants was collected during the follow up period. For the current work depersonalized data of Moscow MONICA were accessed June 1^st^, 2023.

#### RusFMD

For country-level statistics for 1988–2019 the data from RusFMD [11] were used. They were provided by the Center for Demographic Research of the Russian School of Economics and contain detailed indicators of birth rates and mortality of the population of Russian regions. All descriptive statistics presented in RusFMD were calculated on the basis of population statistics data received from the Federal State Statistics Service.

## Methods

In the classical setting of survival analysis, each patient may be assigned an "event" or "censored" status. It is generally assumed that censoring does not contain any information about the timing of a potential event. For ESSE-RF this assumption is not entirely valid in the following sense. Due to the difficulty of accessing personal data of ESSE-RF participants, some events of interest among the participants could have not been recorded. Namely, in some considerable number of cases ESSE-RF investigators were unable to contact either the participant or their representatives ever since the potential event had occurred to the participant. According to the established methodology of epidemiological studies, all such observations were logged into ESSE-RF as censored from the date of the last contact.

For every censored observation in ESSE-RF it is impossible to say whether it was an actual non-informative censoring or a censoring within the problem identified in the previous paragraph. However, it was clear that the “censored” observations described above could not be processed regularly. We would refer to the phenomenon described in the paragraphs above as to “event information loss”.

### Assessment of cardiovascular events loss in ESSE-RF

SCORE2 provides an estimate of a CV event occurrence risk. Therefore, to validate SCORE2 risk estimates using the CV event incidence among ESSE-RF participants, event information losses should be accounted for. It was assumed that information about primary CV events in ESSE-RF was lost with the same probability as information about deaths. This assumption would allow to take the estimate of the mortality information loss for the estimate of the primary CV event information loss.

It may seem that mortality information loss could be estimated via comparison of mortality rates in ESSE-RF and Russian Fertility and Mortality database. However, mortality rates in ESSE-RF would a priori differ from the mortality rates of the entire Russian population due to inclusion criteria of the study [9]. For example, subjects from marginal strata of the population, people with terminal stages of diseases or people with a high level of distrust in scientific research were not included in ESSE-RF. Therefore, a direct comparison of mortalities in ESSE-RF and RusFMD would not be meaningful.

To estimate the mortality information loss in ESSE-RF using RusFMD, it was necessary to estimate the discrepancy between mortality rates in the entire Russian population and mortality rates in the Russian population meeting ESSE-RF inclusion criteria. This discrepancy would be called as the representativeness coefficient of ESSE-RF relative to the entire Russian population and denoted by the coefficient *C*. The exact interpretation of *C* is as follows: let *C* be equal to 2, then in the entire Russian population, the mortality rate is twice as high as in the Russian population meeting ESSE-RF inclusion criteria.

The representativeness coefficient of ESSE-RF study was estimated using Moscow MONICA, since it was assumed that ESSE-RF and Moscow MONICA had the same representativeness coefficient relative to the corresponding populations. That was because they had the same inclusion criteria and data collection methodology. However, unlike ESSE-RF, all fatal events in Moscow MONICA within the first 7 years of the study were reliably identified because in 1988–1999 the legislation allowed checking the vital status of a person through the registry office. Therefore, all the difference in seven-year mortality in Moscow MONICA and RusFMD data for Moscow, if present, would be explained by the representativeness coefficient of Moscow MONICA relative to the entire population of Moscow. That allowed to estimate representativeness coefficient *C* by comparing mortality in Moscow MONICA and RusFMD.

Since the representativeness coefficient of ESSE-RF was estimated, the mortality information loss could be estimated too. To do so, mortality rates of ESSE-RF were compared with RusFMD mortality rates, adjusted for the representativeness coefficient. Further, the information loss would be denoted by the coefficient *B* and interpreted as follows: let *B* be equal to 1.5, then in the Russian population meeting ESSE-RF inclusion criteria, the mortality was 1.5 times higher than observed mortality in ESSE-RF. Let us note again that mortality information was assumed to be lost with the same probability as information about primary CV events, so the estimate of *B* would be the estimate of primary CV event information loss too. The exact definitions and methods of estimating *C* and *B* are given in the “Statistical analysis” section.

### Methodology of comparison between ESSE-RF cardiovascular event rates and SCORE2 risk estimates

To assess the accuracy of SCORE2 risk estimates for ESSE-RF participants, these estimates were compared with the observed CV event rates of ESSE-RF, adjusted for CV event information loss.

The observed CV event rates of ESSE-RF, adjusted for CV event information loss were calculated as follows. Firstly, we took ESSE-RF data on risk factors and CV events and fitted a model to obtain estimates on 7-year risks of CV events for every ESSE-RF participant. These risk estimates were called the observed CV event rates in ESSE-RF. Secondly, the observed CV event rates were adjusted with the coefficient *B* to account for CV event information loss during the 7-year period. These adjusted rates were considered as the estimates of a CV event risk within 7-years for the ESSE-RF participant from the moment of entering the study. Finally, the 10-year risks of CV events were estimated with the use of risk multiplicativity assumption. 10-year risks of CV events were not estimated directly from ESSE-RF in order to avoid COVID19 pandemic influence on CV event rates. The resulting 10-year risk estimate for every participant would be referred as SCORE2-ESSE. The model is described in more details in statistical analysis section.

The assessment of SCORE2 risk estimates for ESSE-RF participants was made by comparing SCORE2 and SCORE2-ESSE for every participant.

### Statistical analysis

Statistical analysis was performed in R version 4.2.1. Continuous variables were described with median and quartiles: MED [Q25; Q75].

#### Representativeness coefficient

The probabilities of death over a 7-year period for people aged *m* years in an entire population and in a specified part of the population were assumed to relate according to the formula

$$P\left(m<T_{pop}\leq m+7|T_{pop}>m\right)=C\cdot P\left(m<T_{spec}\leq m+7|T_{spec}>m\right),$$

where *T*_*pop*_ denotes a random variable corresponding to the lifetime of a person in the entire population, *T*_*spec*_ denotes a random variable corresponding to the lifetime of a person in a specified part of the population and *C* is a number (does not depend on *m*).

The specified part of the population consisted of those people from the entire population who met the inclusion criteria of the epidemiological study. The coefficient *C* was called the representativeness coefficient of the epidemiological study.

The representativeness coefficient *C* of Moscow MONICA was estimated as follows. The probability on the left side of the formula above was estimated with RusFMD data for the corresponding period; the probability on the right side of the equality was estimated with the MONICA data; *C* was estimated as the ratio of them.

The procedure was carried out for subpopulations aged 40 to 64 years at the beginning of the study (due to data availability and SCORE2 target population criteria) and 25 estimates of the coefficient *C* were obtained:

$$\hat{C_{k}}=\frac{\hat{P}\left(k<T_{pop}\leq k+7|T_{pop}>k\right)}{\hat{P}\left(k<T_{spec}\leq k+7|T_{spec}>k\right)},\;k=40,\ldots 64.$$

The numerator was estimated with a formula analogous to the Kaplan-Meier product limit estimate:

$$\hat{P}\left(k<T_{pop}\leq k+7|T_{pop}>k\right)=1-\left(1-q_{k+6}\right)\ldots \left(1-q_{k}\right),$$

where $q_{k}=\hat{P}(T\leq k+1|T>k)$ were obtained from RusFMD. The denominator was estimated from the MONICA data using classical Kaplan-Meier method. Note that such method for estimating probability in the denominator was valid due to the fact that Moscow MONICA had no event information loss.

The final estimate of the coefficient *C* was the median $\hat{C}=Med\left(\hat{C_{40}},\ldots ,\hat{C_{64}}\right)$. Two estimates of representativeness coefficients were calculated: for men and for women.

As mentioned above, ESSE-RF and MONICA were assumed to have the same representativeness coefficients, therefore after this procedure the estimates of ESSE-RF representativeness coefficients were obtained too.

#### Information loss

The relationship between documented mortality in a study (with mortality information loss present) and mortality in the entire population was assumed to be

$$P\left(m<T_{pop}\leq m+7|T_{pop}>m\right)=C\cdot B\cdot P\left(m<T_{epi}\leq m+7|T_{epi}>m\right),$$

where *T*_*pop*_ denotes a random variable corresponding to the lifetime of a person in the entire population, *T*_*epi*_ denotes a random variable corresponding to a lifetime of a person in a population with the same mortality rates as are documented in the epidemiological study, *C* is the representativeness coefficient, *B* is the mortality information loss (both *C* and *B* do not depend on *m*).


$$B=\frac{P\left(m<T_{pop}\leq m+7|T_{pop}>m\right)}{C\cdot P\left(m<T_{epi}\leq m+7|T_{epi}>m\right)}$$

The coefficient B for ESSE-RF was to be estimated. The probability in the numerator was estimated using RusFMD as described in the previous section. In the denominator the estimate for the representativeness coefficient *C* of ESSE-RF was obtained in the previous section. Then, $P\left(m<T_{epi}\leq m+7|T_{epi}>m\right)$ was estimated with the data of ESSE-RF using the Kaplan-Meier method.

This procedure was carried out for every age from 40 to 64 and the median over these estimates was taken as the final estimate of coefficient B for ESSE-RF.


$$\hat{B_{k}}=\frac{\hat{P}\left(k<T_{pop}\leq k+7|T_{pop}>k\right)}{\hat{C}\cdot \hat{P}\left(k<T_{epi}\leq k+7|T_{epi}>k\right)},\;k=40,\ldots 64,$$

$$\hat{B}=Med\left(\hat{B_{40}},\ldots ,\hat{B_{64}}\right).$$

Under the assumption that mortality information is lost with the same probability as information about CV events, *$\hat{B}$* also estimates the CV event information loss coefficient for ESSE-RF. Two estimates for information loss were calculated: for men and for women.

#### Model

The observed event rates (see definition in the section “Methodology of comparison between ESSE-RF cardiovascular event rates and SCORE2 risk estimates”) were obtained with the use of two Fine-Gray models of competing risks. One model was fitted to the data of ESSE-RF men, another to the data of ESSE-RF women. In both cases the considered participants were those who met the criteria of SCORE2 target population. The criteria were as follows: 40–69 years of age, without CVD and diabetes.

The event of interest was composed of cardiovascular death, nonfatal myocardial infarction (MI) and/or nonfatal acute cerebral circulation disorder. Death from other causes was considered as a competing event.

The list of predictors was identical to the original SCORE2 predicting model: age, systolic blood pressure, HDL, total cholesterol, smoking status, and interactions of these risk factors with age.

The whole procedure was done in accordance with the original SCORE2 article.

#### Adjustment for CV event information loss

In Fine-Gray regression models of competing risks the relationship between a cause specific cumulative incidence function for a particular set of risk factors *F*_*i*_(*t*) and a baseline cumulative incidence function *F*_0_(*t*) is as follows:

$$F_{i}(t)=1-{\left(1-F_{0}(t)\right)}^{\exp\left(\beta x_{i}\right)}$$

where *x*_*i*_ is a vector of risk factors, *β* is a vector of corresponding coefficients.

In case risk factors are normalized, the baseline cumulative incidence function reflects the probability of experiencing the event of interest for an “average” participant of the study. By an average participant is meant a subject whose risk factor levels are averaged among all the study participants.

We then note that according to the section “Information loss” the information loss coefficient is calculated for the whole study (separately for men and women). This implies that the coefficient is intended for adjusting a risk of an average participant of the study. Hence, the adjusted estimate of the risk for an individual with a set of normalized risk factors *x*_*i*_ should be obtained as follows:

$${\hat{F}}_{l}(t)=1-{\left(1-\hat{B}\cdot \hat{F_{0}}(t)\right)}^{exp\left(\hat{\beta }x_{i}\right)},$$

where $\hat{F_{0}}(t)$ is an estimate of the baseline cause specific incidence function and $\hat{\beta }$ is an estimate of *β* derived from the original data.

The effectiveness of the described adjustment procedure is demonstrated by simulation approach in S1 Appendix.

## Results and discussion

In this part, the results of the assessment of SCORE2 risk estimates are provided as well as the estimates of representativeness coefficient and information loss for ESSE-RF. This is followed by the discussion.

### Representativeness coefficient and information loss for ESSE-RF

As it was mentioned before, Moscow MONICA and ESSE-RF were assumed to have the same representativeness coefficients. The estimates calculated from Moscow MONICA and RusFMD data were equal to 1.63 for men and to 1.74 for women.

Estimates of ESSE-RF CV event loss coefficients were obtained in accordance with “Information loss” section and were equal to 1.53 for men and to 1.44 for women. These coefficients meant that information about approximately every third primary CV event in ESSE-RF was lost.

### Accuracy assessment of SCORE2 risk estimates for ESSE-RF participants

**Fig 1:**
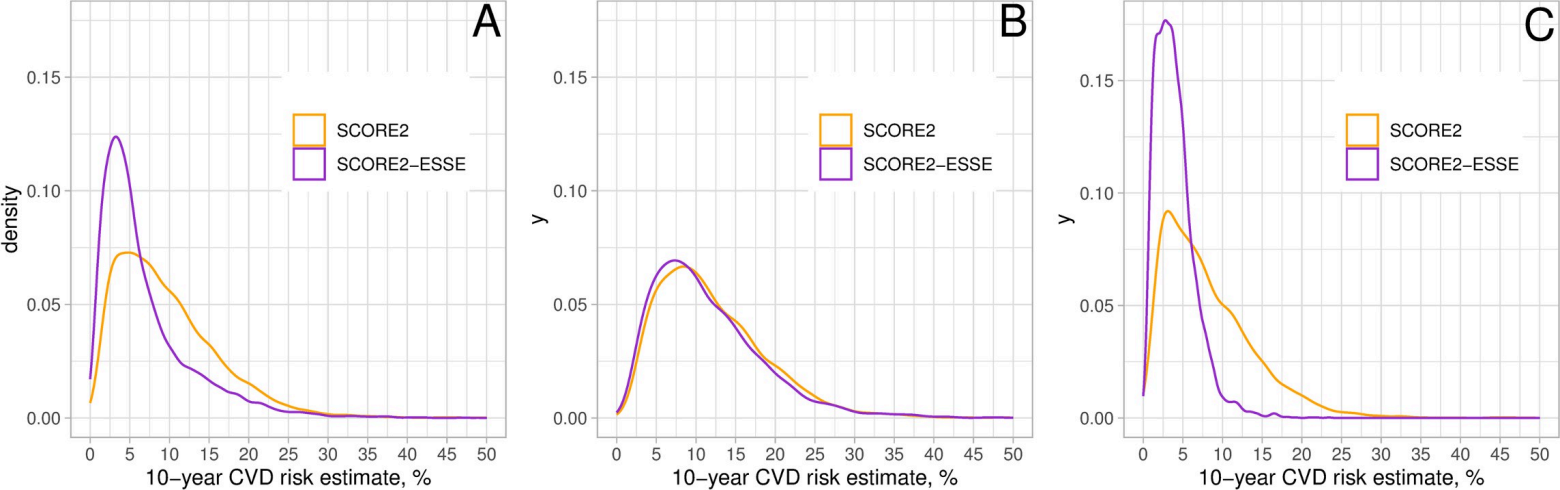
Distribution of SCORE2 and SCORE2-ESSE for ESSE-RF participants who meet SCORE2 target population criteria. A: all; B: men; C: women.

To begin the assessment, distributions of SCORE2 and SCORE2-ESSE were compared. Fig 1 presents the distributions of SCORE2 and SCORE2-ESSE for those ESSE-RF participants who met SCORE2 target population criteria.

**Fig 2:**
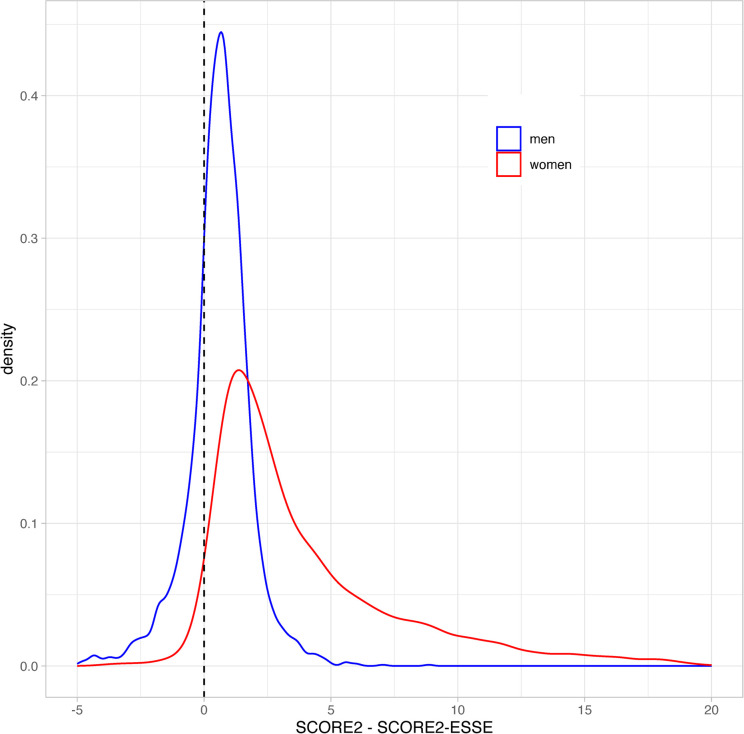
Distribution of the difference between SCORE2 and SCORE2-ESSE for ESSE-RF participants.

Fig 2 presents the distribution of the difference between SCORE2 and SCORE2-ESSE separately for men and for women.

The difference between SCORE2 and SCORE2-ESSE were equal to 0.7 [0.0, 1.3] for men, and 2.6 [1.3, 5.6] for women. The quantiles of absolute difference between SCORE2 and SCORE2-ESSE were 0.9 [0.4, 1.5] for men and 2.7 [1.3, 5.6] for women.

For men descriptive statistics on SCORE2 and SCORE2-ESSE suggested that the level of consistency between the two scales was high. That was displayed by the coincidence of SCORE2 and SCORE2-ESSE density functions, and concentration of distribution of the difference between SCORE2 and SCORE2-ESSE around zero. For women the consistency of the scales was significantly lower. In particular, the overestimation of SCORE2 over SCORE2-ESSE for women was detected via the distribution of the difference between SCORE2 and SCORE2-ESSE. The detected heterogeneity inferred that the SCORE2 risk estimates should be further assessed separately for men and for women.

**Fig 3:**
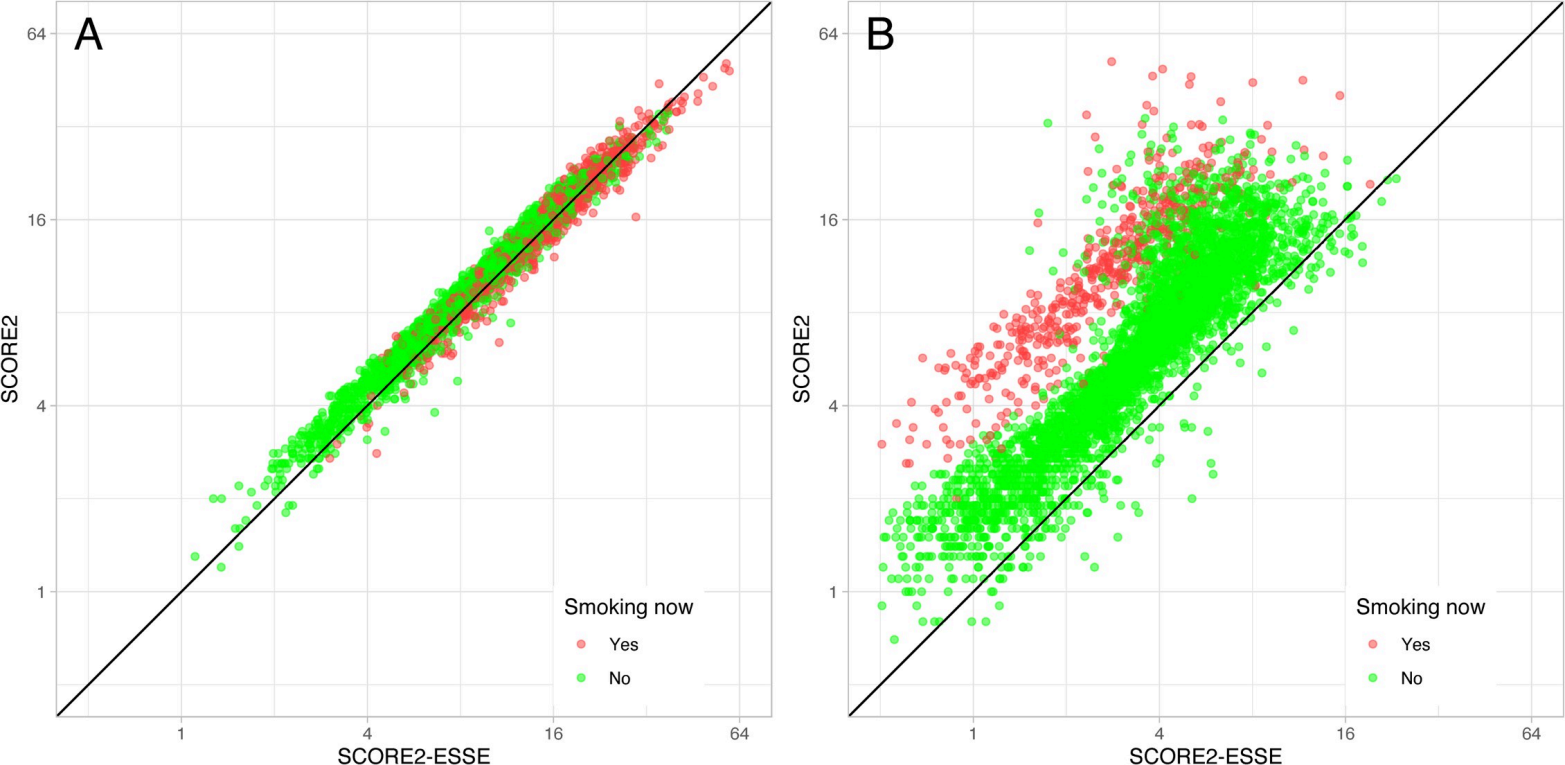
Correspondence of SCORE2 and SCORE2-ESSE. A: men; B: women. The diagonal line shows where SCORE2 and SCORE2-ESSE match.

Fig 3 shows the correspondence of SCORE2 and SCORE2-ESSE for ESSE-RF participants. For men (Fig 3A), the plot supported the idea of the consistency between SCORE2 and SCORE2-ESSE. The coefficients of mean calibration [12] between SCORE2 and SCORE2-ESSE risks for men were 1.004 for slope (p = 0.20 for comparison with 1) and 0.738 for intercept (p<0.001 for comparison with 0). This meant that the estimates for men were consistent and differed by a small constant value for the entire risk range. For women (Fig 3B), the overestimation of SCORE2 over SCORE2-ESSE was detected once again. The coefficients of mean calibration between SCORE2 and SCORE2-ESSE risks for women were 1.507 for slope (p<0.001 for comparison with 1) and 2.193 for intercept (p<0.001 for comparison with 0). Another aspect identified by the plot was the clustered structure of the points by smoking status for women. That implied the difference between the coefficients for smoking status in SCORE2 and SCORE2-ESSE models for women and confirmed the discrepancy between SCORE2 and SCORE2-ESSE for women.

**Fig 4:**
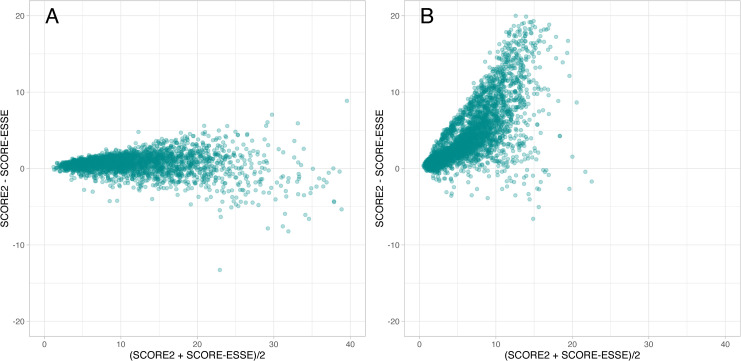
Bland-Altman plot of SCORE2 and SCORE2-ESSE risk estimates. A: men; B: women.

The consistency between SCORE2 and SCORE2-ESSE for men was also confirmed by the Bland-Altman plot, since the graph had the form of a narrow isosceles triangle symmetrical with respect to zero (Fig 4A). For women, in turn, the asymmetrical Bland-Altman triangle confirmed the overestimation of SCORE2 over SCORE2-ESSE (Fig 4B).

Based on the information given in the section, we concluded that SCORE2 and SCORE2-ESSE were consistent for men participating in ESSE-RF. This meant that SCORE2 risk estimates for men participating in ESSE-RF were accurate. From that we derived that SCORE2 is an accurate predictive instrument for the population of Russian men.

In contrast, we showed the inconsistency between SCORE2 and SCORE2-ESSE for women participating in ESSE-RF and therefore concluded that SCORE2 cannot be used as a predictive instrument for the population of Russian women. We will investigate this problem outside the scope of this work.

### Suggestions for adaptations of SCORE2 for clinical practice

This section presents an adapted interpretation of SCORE2 risk estimates for clinical practice in Russia and other countries of very high risk region. Since we agreed on SCORE2 giving accurate risk estimates for Russian men but not for Russian women, a meaningful interpretation of SCORE2 risk estimates for Russian population is only possible for men. Therefore, in this section only the population of Russian men would be considered.

Along with CV event risk estimates, SCORE2 has an interpretation in terms of 10-year CVD risk groups: low-to-moderate risk group, high risk group or very high risk group. In the men subgroup aged 40 to 49 years, the cutoff points for the risk groups were 2.5 and 7.5%. That is, men with SCORE2 of less than 2.5% were considered to be at low-to-moderate risk, those with SCORE2 between 2.5% and 7.5% were considered to be at high risk, and those with SCORE2 above 7.5% were considered to be at very high risk of CV event within 10 years. In the 50 to 69 year old subgroup, the corresponding cut-off values were 5 and 10%. From now on, these cutoff values would be referred as to original cutoff values or original cutoffs.

**Fig 5:**
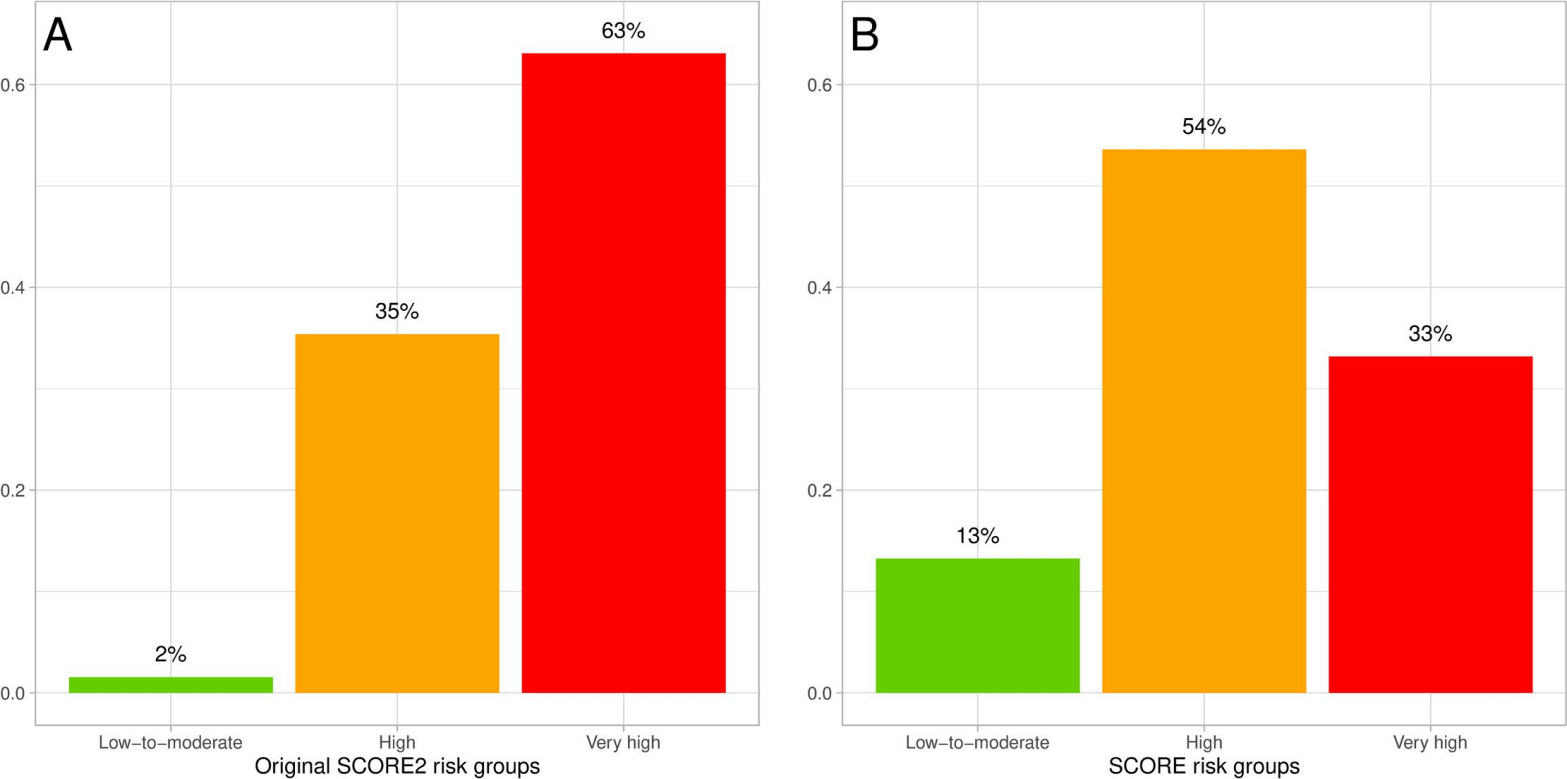
Distribution of ESSE-RF participating men. A: into SCORE2 risk groups of original cutoff values; B: into SCORE risk groups.

The original cutoff values were considered questionable for two main reasons. The first reason was that according to the original cutoff values, 63% of men in ESSE-RF were assigned to a very high 10-year CVD risk group (Fig 5A). From that it could be approximated that 2 of 3 Russian men would be considered at “very high” 10-year CVD risk and according to ESC guideline would be generally recommended CVD risk factor treatment. Such strategy would paralyze the public healthcare system. To compare, according to SCORE, 33% of men in ESSE-RF were considered at very high risk (Fig 5B).

The second reason was that the distribution of men into risk groups defined by the original cutoff values was explained more by high baseline risks of CV events in Russia than by levels of the risk factors of the participants. This can be illustrated with the following example.

*Example*. A French (representative of the low risk region), a German (representative of the moderate risk region), and a Russian (representative of the very high risk region) men aged 51 years with the same SCORE2 risk factor values of current smoking, SBP 125, total cholesterol 4.8 and HDL cholesterol 1.2 visited a cardiologist. According to SCORE2 they would be assigned a risk of having a CV event within 10 years of 4.9%, 6.2%, and 12%, respectively. Based on the original cutoff values, the French would be at low-to-moderate risk, the German would be at high risk, and the Russian would be at very high risk of CV event within 10 years. This implies that according to ESC guidelines these three men would have different recommendations on further prevention and treatment of the SCORE2 risk factors. This is a questionable result given that their SCORE2 risk factors are identical. In our opinion, this situation would be incorrect from the point of view of preventive medicine and indicates the need of determining different cutoff values for each risk region.

To approach the problem illustrated in the example, we proposed to define cutoff values for each risk region separately as follows. For a hypothetical individual, it is possible to observe how his SCORE2 risk estimate will change if the risk factor values are fixed, and the risk region is changing. In the example considered, a risk of 4.9% in the low risk region would correspond to a risk of 6.2% in the moderate risk region and 12% in the very high risk region.

Cutoff values for the very high CVD risk region were calculated using the correspondence between the very high CVD risk region and the moderate CVD risk region. The moderate risk region was taken as a reference mainly because the resulting distribution of ESSE-RF men into CVD risk groups resembled the distribution given by SCORE. According to the procedure described in the previous paragraph, original cutoff value of 2.5% risk for the moderate risk region converted into 4.5% for the very high risk region. Similarly, the conversions were 5% into 9%, 7.5% into 14% and 10% into 18%.

The converted version of original cutoff values is proposed to be used in the countries of the very high CVD risk region (Table 1).

**Table 1:** Proposed cutoff values for men in a very high risk region.

	<50 years	50–69 years
Low-to-moderate CVD risk: risk factor treatment generally not recommended	<4.5%	<9%
High CVD risk: risk factor treatment should be considered	4.5%-14%	9%-18%
Very high CVD risk: risk factor treatment generally recommended	≥14%	≥18%

Table 1 could be referred to as an adapted interpretation of SCORE2 risk estimates for countries of the very high risk region.

**Fig 6:**
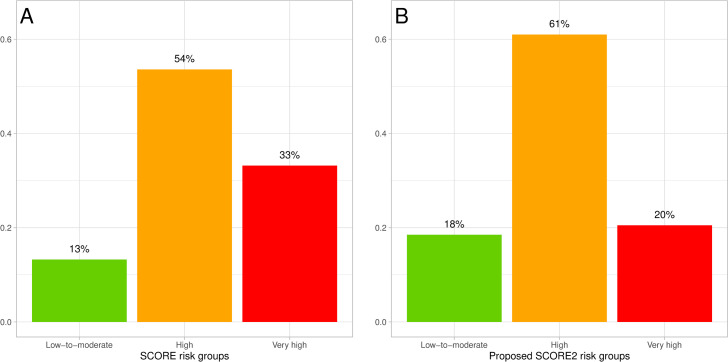
Distribution of ESSE-RF participating men. A: into SCORE risk groups; B: into SCORE2 risk groups of proposed cutoff values.

The distribution of ESSE-RF men into risk groups defined by the proposed cutoff values was as follows (Fig 6A). For reference, the distribution of men by risk groups according to SCORE is presented again (Fig 6B).

**Fig 7:**
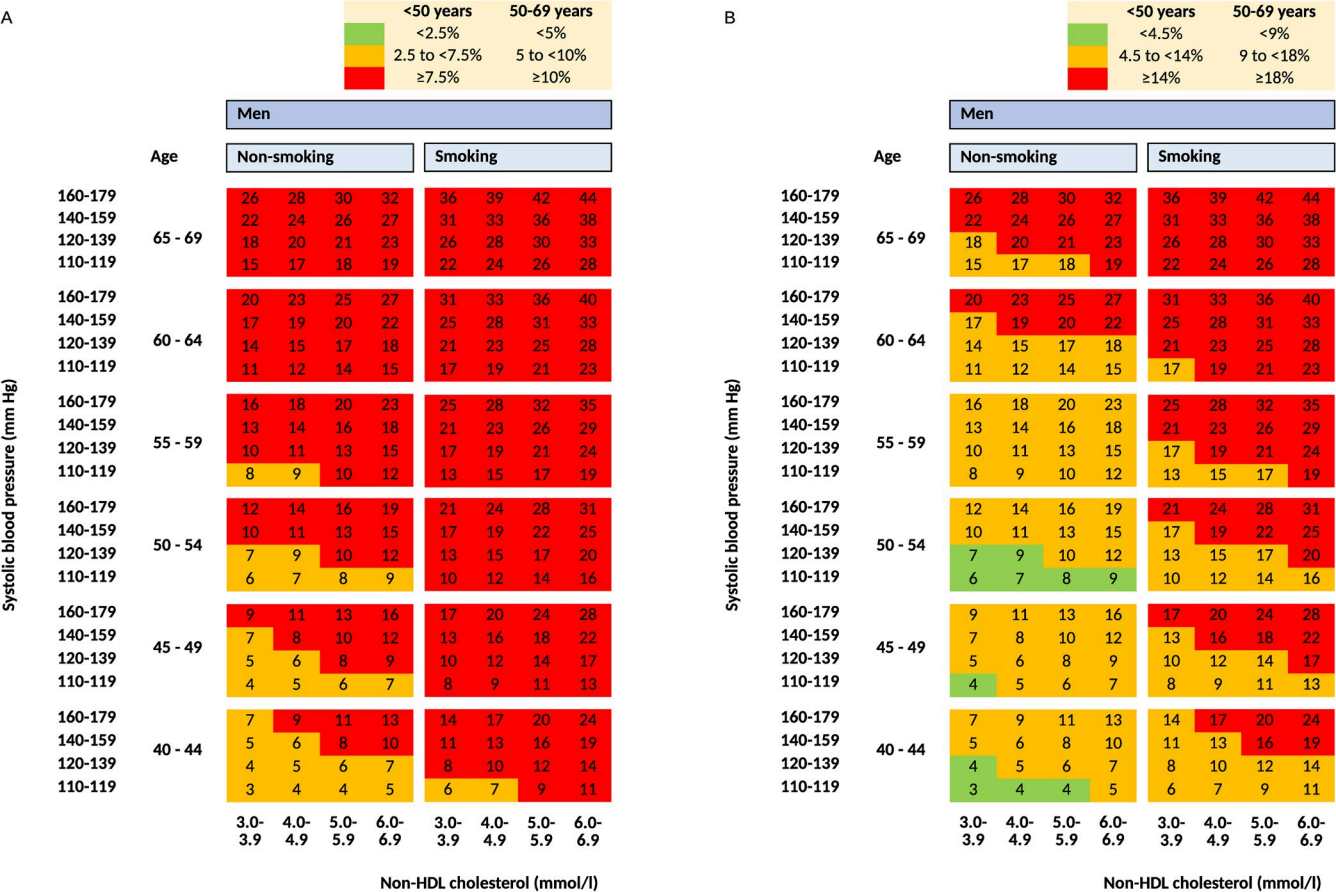
Chart for calculating SCORE2. A: with original coloring; B: with proposed coloring.

The chart for calculating SCORE2 from the original article is presented (Fig 7A). We present a recolored version of the chart to be used in the population of men in Russia and other countries of the very high CVD risk region (Fig 7B). The new coloring is defined by the proposed cutoff values.

We note separately that the methodology presented in the paper would allow validation of any other risk scale for the Russian population such as, for example, American College of Cardiology/American Heart Association [13]. In turn, different validated risk scales would enable a comparison of treatment strategies based on SCORE2 with the treatment strategies based on other commonly utilized risk assessment tools. Such comparisons would provide a more complete understanding of SCORE2 as well as its strengths and limitations, thereby improving practical usefulness.

## Discussion

SCORE2 was developed for more effective identification of individuals with increased risk of CVD. This scale considers the impact of competing risks by non-CVD deaths and provides risk estimates for the combined outcome of fatal and non-fatal CVD event [2]. However, SCORE2 has one potential limitation for countries of the very high CVD risk region. The SCORE2 model was trained and validated on cohorts mostly in European regions and populations at low or moderate risk of CVD, whereas representative data from high and very high risk regions were not used [2]. Kasim S.S. et al. considered the use of SCORE2 for Asian region and indicated that SCORE2 risk model would need to be recalibrated before it could be employed in a different population [14]. We decided to validate SCORE2 for the Russian population using data from the ESSE-RF epidemiological study. The results demonstrated that SCORE2 risk estimates were accurate for Russian men. Hence, the recalibration of SCORE2 before the use in the population of Russian men is not needed. At the same time, SCORE2 risk estimates for Russian women turned out to be overestimated as well as some of the risk factors included in the SCORE2 model were misadjusted. It was supposed that the inaccuracy of SCORE2 risk estimates for Russian women was related to the difference in patterns of the risk factors and their impact to morbidity and mortality between European and Russian women [15]. Therefore, SCORE2 cannot be considered as an accurate predictive instrument for Russian women and will be further investigated outside the scope of this paper.

SCORE2 was included in the ESC Guidelines for Cardiovascular Disease Prevention in Clinical Practice 2021, that provided preventive recommendations at the population and individual level. At an individual level, the guideline provided a step-by-step approach to lifestyle modification and appropriate prescription of treatment based on SCORE2 risk estimate and comorbidities [16]. For example, treatment of atherosclerotic CVD risk factors was recommended in apparently healthy people with SCORE2≥7.5% for age under 50 and SCORE2≥10% for age 50–69 [16].

Since the publication of the guidelines, there have been numerous articles discussing the need of the replacement of SCORE with SCORE2. Researchers were dissatisfied with the fact that based on SCORE2, a significant proportion of the population is considered at the very high risk instead of the previous low–moderate or high risk according to SCORE. That would lead to the unmanageable load in the primary healthcare systems of the countries of SCORE2 very high risk region [17]. In our study, an enormous fraction of 63% of ESSE-RF men among those who met SCORE2 target population criteria were classified into a very high 10-year CVD risk group.

The problem outlined in the paragraph above formed the objective of modifying the interpretation of SCORE2 risk estimates for the countries of very high risk region. As a result of mathematical modeling the adapted interpretation of SCORE2 risk estimates for men was proposed. It was suggested to attribute men from very high risk region to the group of very high 10-year CVD risk with SCORE2 ≥14% (<50 years) and ≥18% (50–69 years). In accordance with the adapted interpretation the fraction of men in ESSE-RF in “low-to-moderate” 10-year CVD risk increased from 2% to 18% the fraction of men in ESSE-RF with very high CVD risk dropped from 63% to 20%.

## Limitations

To obtain adjusted CV event rates a set of assumptions was made. First, it was assumed that both representativeness and information loss coefficients were constant across age groups. Second, it was assumed that information about CV events was lost with the same probability as information about deaths. Third, a risk multiplicativity assumption was used to derive estimates of 10-year risk from estimates of 7-year risk. We considered these assumptions to be logically valid; however, since they could not be formally verified from the data, they are mentioned as limitations of the study.

Another limitation is related to the recolored version of the SCORE2 calculation chart for men in the very high CVD risk region. In clinical practice of a particular country of the very high CVD risk region, the chart could be used only upon the validation of SCORE2 in its population. In other words, SCORE2 risk groups could only be meaningful if the accuracy of SCORE2 risk estimates for the particular population was assessed.

## Conclusions

The accuracy of SCORE2 for the population of Russian men was proved. SCORE2 for Russian women was proved to be inaccurate.

The adapted interpretation of SCORE2 for men populations of Russia and other countries of very high risk region was proposed. According to the interpretation, the fraction of men in ESSE-RF in “low-to-moderate” 10-year CVD risk increased from 2% to 18% and the fraction of men in “very high” CVD risk decreased from 63% to 20% as compared to the original interpretation. The proposed interpretation would allow a more personalized approach to CVD treatment and optimize the burden on primary healthcare in the very high risk region countries.

## Supporting information

S1 Appendix (DOCX)

S1 Table Simulated data for the number of CVD events after loss, the number of competing events after loss and the loss coefficient estimate. (XLSX)

S2 Table Calibration coefficients for adjusted and unadjusted estimates. (XLSX)

S1 Data (DOCX)
