# Supplementary material for: Validation of SCORE2 on a sample from the Russian population and adaptation for the very high cardiovascular disease risk region
Source: PLoS One. 2024 Apr 17;19(4):e0300974. doi: 10.1371/journal.pone.0300974 (PMC11023576; doi:10.1371/journal.pone.0300974)
Supplement: S1 Appendix — (DOCX) [file pone.0300974.s001.docx]

# S1 Appendix

To demonstrate the effectiveness of the adjustment method a simulation approach was used. We generated 100 copies of data such that after a simulated information loss (described below) the data would be similar for ESSE-RF men meeting SCORE2 inclusion criteria. Specifically: 2850 participants, 7-year follow-up period, approximately 150 CV events (including CVD deaths) and approximately 85 competing events (non-CVD deaths).

Competing risks data were modeled under the assumption of proportional subdistribution hazard functions using R adjustedCurves package[1]. To model effects of risk factors on CV events coefficients from the original SCORE2 article were used.

The information loss was simulated with accordance to the definition of the information loss given in the beginning of the “Methods” section. That is, a selected percentage $\gamma$ of all “events” was replaced by the status “censored”, and the time of those censored events was set to be:

$$t_{cens}=t_{event}-Unif\left( 0,2 \right),$$

where $Unif\left( 0,2 \right)$ is a realization of a random variable with uniform distribution with a support [0,2]. That was done in such a way because in ESSE-RF the vital status of every participant was checked once in two years. Consequently, to simulate the loss of a specific event a random number between 0 and 2 should be subtracted from the event date.

By this point, for every simulated dataset we had a set of events and a set of times before and after performed information loss. The information loss coefficient was then estimated as described in the “Methods” section, that is, by comparing survival curves for fatal events at the 7-years observation point before and after loss:

$$\hat{B}=\frac{7year KM estimate based on the original data}{7year KM estimate based on the data after loss}.$$

The percentage $\gamma$ defined above was chosen such that $\hat{B}$ would be approximately 1.53 (the actual estimate of the information loss coefficient for ESSE-RF men). The simulated data for the number of CVD events after loss, the number of competing events after loss and the loss coefficient estimate for each of the 100 simulations are presented in S1 Table.

To conclude on the effectiveness of the adjustment method we compared adjusted and unadjusted post-loss risk predictions with pre-loss risk predictions. Note, that the pre-loss risk predictions represented the classic Fine-Gray approach to predicting cause-specific risks in a competing risk setting.

To compare pre-loss predictions (unadjusted and adjusted) with post-loss predictions weak calibration was used. For the unadjusted predictions calibration coefficients were 1.27 for slope (p < 0.001 for comparison with 1) and 0.70 for intercept (p = 0.003 for comparison with 0). In turn, for the adjusted predictions calibration coefficients were 0.99 for slope (p = 0.375, for comparison with 1) and -0.04 for intercept (p = 0.84 for comparison with 0). That directly implied that the adjustment procedure is effective in a sense that it provided unbiased 7-year risk estimates constructed from the post-loss data, whilst unadjusted estimates were biased (underestimated risk).

Data on calibration coefficients for adjusted and unadjusted estimates is provided in S2 Table.

# References

1. Denz R, Klaaßen-Mielke R, Timmesfeld N. A comparison of different methods to adjust survival curves for confounders. Stat Med. 2023;42: 1461–1479. doi:10.1002/sim.9681
