# Supplementary material for: Validation of SCORE2 on a sample from the Russian population and adaptation for the very high cardiovascular disease risk region
Source: PLoS One. 2024 Apr 17;19(4):e0300974. doi: 10.1371/journal.pone.0300974 (PMC11023576; doi:10.1371/journal.pone.0300974)
Supplement: S1 Data — (DOCX) [file pone.0300974.s004.docx]

Data of ESSE-RF and Moscow MONICA cannot be shared publicly in accordance with the rules of the ethics committee of the National Medical Research Center for Therapy and Preventive Medicine regulations. Deidentified data will be provided on reasonable request to the corresponding author (gleb.e.svinin@gmail.com) or to the Research Assistant of the Department of Epidemiology of Non-communicable Diseases, Mr. Oleg Ivlev (olivlegerr@gmail.com). Proposals will be reviewed and approved by the researchers, local regulatory authorities, and the ethics committee of the National Medical Research Center for Therapy and Preventive Medicine. Once the proposal has been approved, data may be transferred through a secure online platform after signing a data access agreement and a confidentiality agreement.
